# Automatic classification of written descriptions by healthy adults: An overview of the application of natural language processing and machine learning techniques to clinical discourse analysis

**DOI:** 10.1590/S1980-57642014DN83000006

**Published:** 2014

**Authors:** Cíntia Matsuda Toledo, Andre Cunha, Carolina Scarton, Sandra Aluísio

**Affiliations:** 1 Faculdade de Medicina da Universidade de São Paulo, SP, Brazil.; 2 Núcleo Interinstitucional de Linguística Computacional (NILC). Instituto de Ciências Matemáticas e de Computação – Universidade de São Paulo, SP, Brazil.; 3 Department of Computer Science, University of Sheffield - Sheffield, UK.

**Keywords:** natural language processing, language tests, narratives, adults, educational status, age groups

## Abstract

Discourse production is an important aspect in the evaluation of brain-injured individuals. We believe that studies comparing the performance of brain-injured subjects with that of healthy controls must use groups with compatible education. A pioneering application of machine learning methods using Brazilian Portuguese for clinical purposes is described, highlighting education as an important variable in the Brazilian scenario.

**Objective:**

The aims were to describe how to:

(i) develop machine learning classifiers using features generated by natural language processing tools to distinguish descriptions produced by healthy individuals into classes based on their years of education; and
(ii) automatically identify the features that best distinguish the groups.

**Methods:**

The approach proposed here extracts linguistic features automatically from the written descriptions with the aid of two Natural Language Processing tools: Coh-Metrix-Port and AIC. It also includes nine task-specific features (three new ones, two extracted manually, besides description time; type of scene described – simple or complex; presentation order – which type of picture was described first; and age). In this study, the descriptions by 144 of the subjects studied in Toledo^18^ were used,which included 200 healthy Brazilians of both genders.

**Results and Conclusion:**

A Support Vector Machine (SVM) with a radial basis function (RBF) kernel is the most recommended approach for the binary classification of our data, classifying three of the four initial classes. CfsSubsetEval (CFS) is a strong candidate to replace manual feature selection methods.

## Abstract (pt)

Um importante aspecto na avaliação de indivíduos com lesão cerebral é a produção de discurso. Acreditamos que estudos que comparam o desempenho de lesados com grupos de controles sadios devem utilizar grupos com escolaridade compatíveis. Nós apresentamos uma abordagem pioneira ao utilizar métodos de aprendizado de máquina com propósitos clínicos, para o Português do Brasil, destacando a escolaridade como variável de importância no cenário brasileiro.

**Objetivo:**

Nosso objetivo é descrever como:

(i) desenvolver classificadores via aprendizado de máquina, usando features criadas por ferramentas de processamento de línguas naturais, para diferenciar descrições produzidas por indivíduos sadios em classes de anos de escolaridade e
(ii) identificar automaticamente as features que melhor distinguem esses grupos.

**Métodos:**

A abordagem proposta neste estudo extrai características linguísticas automaticamente a partir das descrições escritas com a ajuda de duas ferramentas de Processamento de Linguagem Natural: Coh-Metrix-Port e AIC. Ela inclui ainda nove features dedicadas à tarefa (três novas, duas extraídas manualmente, além de tempo de descrição; tipo de cena descrita - simples ou complexa; ordem de apresentação das figuras e idade). Neste estudo, foram utilizadas as descrições de 144 indivíduos estudados em Toledo^18^, que incluiu 200 brasileiros, sadios, de ambos sexos.

**Resultados e Conclusão:**

SMV com kernel RBF é o mais recomendado para a classificação binária dos nossos dados, classificando três das quatro classes iniciais. O método de seleção das features CfsSubsetEval (CSF) é um forte candidato para substituir métodos de seleção manual.

## INTRODUCTION

Discourse has been considered an essential and discriminating element to interpret language evaluations.^1^ A wide variety of discourse types and measures has been investigated, an interest influenced by the acknowledgment that discourse is a natural form of communication, and may provide important information about linguistic micro and macrostructures^2^ and about the integration of linguistic and cognitive skills.^3,4^

The narrative discourse elicited by pictures is useful for research, since it brings out speech in a standardized way and allows for comparison between individuals and groups.^5^

In studies of brain-injured patients, the research subjects have included individuals with focal lesions,^2^ diffuse lesions, and degenerative processes.^6^ Most research compares the performance of brain-injured individuals with that of healthy subjects.^7^ Little emphasis has been given to discourse production in normal individuals. Characterizing the performance of these individuals may help diagnosing, evaluating, and rehabilitating subjects with language impairments.

An important justification for the study of normal individuals is the wide variety of discourse production foreseen in the task. Among the causes for such diversity are age and education. Many studies refer to age effects on the length of the material produced, on information content, coherence, and fluency of statements.^3,8,9^ The discourse of more highly educated individuals has been reported as being longer and more dense in content.^10-12^ Education influences lexical decision-making ability, phonological knowledge, and visuospatial abilities.^13,14^

Data on the characteristics of the adult population's discourse are limited. In the absence of a reference framework for the normal population, clinicians evaluate their patients' performance based on subjective, variable criteria. Most studies analyze data manually. Notable among these investigations is the research by Mackenzie,^11^ Marini et al.,^8^ Forbes-McKay and Venneri,^15^ Alves and Souza,^16^ Parente et al.,^17^ and Toledo,^18^ which is of special interest to this work.

Recent years have been marked by advances in both the compiling and sharing of discourse samples (*cf*., for example, the TalkBank project – http://talkbank.org/ – that provides databases for investigations on aphasia and dementia), as well as the use of Natural Language Processing (NLP) methods to analyze the written discourse of brain-injured patients and their healthy controls.^19,20^ Such initiatives have allowed the development of systems to access and share human language data,^21^ along with methodological improvements on discourse analysis. In the study of Fraser et al.,^19^ automatic methods for extracting linguistic features from narrative transcripts were used, with important interventions in the transcripts, to generate good-performance classifiers.

NLP technologies can improve language analyses and samples considerably, and change clinical practice through quantifiable measures not affected by human subjectivity or by the lack of uniformity in manual annotation.^22^ NLP methods can be used to quantify and describe language difficulties in natural contexts, and also allow for improving comparative analyses before and after therapeutic interventions for language rehabilitation.

Researchers at the University of Memphis developed Coh-Metrix,^23^ a tool to calculate text cohesion and difficulty using several levels of analysis. Coh-Metrix 2.0 is the freeware version of this tool, with 60 metrics from psycholinguistics and NLP. This tool was adapted to Brazilian Portuguese (BP) as Coh-Metrix-Port,^24^ with 48 metrics to analyze lexical, morphosyntactic, nounphrase-level syntactic, semantic, and discourse features.^25^ In addition, the PorSimples project –nilc.icmc.usp.br/porsimples/index.php/English – developed a tool called AIC dedicated to text analysis emphasizing syntactic measures derived from full parsers, making up for the lack of this level of analysis in Coh-Metrix-Port.

In the study by Cunha et al.,^26^ we used Coh-Metrix-Port and AIC to analyze discursive tasks in BP involving healthy individuals. Also regarding BP, we have found no investigation comparing brain-injured subjects and their healthy controls using NLP approaches. The aforementioned study is a first step in the development of a computational environment called Coh-Metrix-Dementia, aimed at automatically extracting several features from speech transcripts in BP, and intended to provide clinical analyses instruments to differentiate healthy adults from individuals with different types of dementia.

In this paper, we take as an example our proposal by Cunha et al.^26^ to spread the use of both the Machine Learning (ML) approach and NLP tools to automate clinical discourse analysis. ML is a set of techniques intended to endow computers with the ability to perform tasks without being explicitly programmed. Supervised ML consists of using a set of labeled examples, called the *training set*, to build a predictive model, capable of analyzing a previously unseen example and assigning it a label. If the labels are in fact classes to which the samples belong, the task is called *classification*. In the training set, each example is described by a series of *features* (also called *attributes*), which the ML algorithm uses to generate the model.

The task explored here is to develop classifiers to tell apart descriptions by individuals with different years of education. Education was chosen among the three sociodemographic variables evaluated (education, gender, and age) because it showed a stronger influence in Toledo's study,^18^ whose data is used here (see the section below). In particular, the aim is to explain the difficulties in preparing the data to be used with NLP tools and in choosing the classes (years of education), along with possible solutions for classifying our data, and lessons learned in the computational treatment of discourse in the clinical context of brain-injured patients' evaluations, which involve the discourse of healthy controls. This study's research questions are as follows:

- Is there a more appropriate multiclass ML method to distinguish picture descriptions written by groups of healthy individuals based on education? If yes, which number of classes would allow for a better performance?
- Can automated feature selection methods (explained later on) retrieve the features (or their automatic equivalents) proved statistically significant in the literature on traditional analyses? Which of these methods retrieve features that generate the best performing classifier?

## MACHINE LEARNING MATERIALS AND METHODS

The approach described here extracts linguistic features automatically from the written descriptions with the aid of Coh-Metrix-Port – http://www.nilc.icmc.usp.br/porsimples/coh_metrix_port/ and AIC – nilc.icmc.usp.br/porsimples/AIC/, and uses them to train classifiers to predict a subject's number of years of education based on their description.

Nine task-specific features were included (three new ones, two extracted manually, besides description time; type of scene described – simple or complex; presentation order – which type of picture was described first; and age); the last six were based on Toledo.^18^

**Participants.** In this study, the descriptions by 144 of the subjects studied in Toledo's project^18^ were used, which was reviewed and approved by the Ethics Committee of the Faculty of Medicine, University of São Paulo (CAPPesq) (Proc. n. 0544/09). The subjects were chosen from among patients' caregivers in the *Ambulatório de Geriatria Cognitiva do Hospital das Clínicas da Faculdade de Medicina da Universidade de São Paulo-HCFMUSP*. All subjects signed a Free and Informed Consent form. The author defined the following inclusion criteria: aged 30 years or older; the MOANS (Mayo Older American Normative Studies) criteria;^7^ Brazilian Portuguese as the first language; three or more years of education; ability to write a sentence (evaluated according to the item of the Mini-Mental State Examination-MMSE that asks to write a sentence); and absence of auditory and visual complaints.

This project was chosen because it is one of the Brazilian studies that involves manual data analysis to which we had access. Toledo^18^ worked with 200 subjects; however, 56 participants were excluded in the present study because their discourses were too short (comprising only a title or a list of words about the pictures) or consisted of personal judgments instead of picture descriptions, which was the proposed task. More details for excluding individual descriptions (and, consequently, individuals themselves) can be found in the next section.

**The picture description task and the research corpus.** Evaluations were carried out individually, with an average duration of 30 minutes. Two pictures were used, each depicting a different scene (one simple and one complex). The simple picture depicts a domestic scene, "A woman tripping up",^27^ whereas the complex picture depicts a traffic scene, "Traffic chaos" (unknown author). Each subject was instructed to write down all he/she could observe in each picture. To minimize the effects of memory difficulties, the pictures were displayed until the subject ended the task.

Toledo^18^ worked with 400 descriptions. We excluded 158 descriptions from various groups. Exclusion was highest among descriptions by participants with 3 to 4 years of education. Descriptions by individuals with more education (especially those with 15 years or more) also have an impact on the automatic analysis, for they include analogies (for example: "Final de tarde"/"End of afternoon"; "São Paulo às 18:00 horas"/"São Paulo at 6 PM"), judgments ("Falta de atenção"/"Lack of attention"; "A falta de humanidade de ajudar o próximo"/"Lack of humanity to help the neighbor"; "A intolerância de uns com os outros"/"Intolerance of one with the others"; "Família meio viciada"/"Somewhat addicted family"), lists of simple observations about the pictures ("Centro, avenida, congestionamento"/"Downtown, avenue, traffic jam"; "Estresse, caos"/"Stress, chaos"), and titles that summarize the picture ("Caos urbano"/"Urban chaos"; "Confusão no trânsito"/"Confusion in the traffic"; "O estress do dia a dia"/"Daily stress"). Because they are short, these texts pose difficulties for the automatic analysis by computer tools and become similar to descriptions by individuals with less education,^10,11^ impairing classification. These discrepancies led to the exclusion of the descriptions that did not comply with the most prototypical form of the task, which includes the construction of a descriptive narrative of the figure in detail. The use of only a title to describe a picture or personal judgments in the place of a description was never intended.

The remaining 242 descriptions were divided as follows: 43 descriptions by participants with 3 to 4 years of education; 64 by participants with 5 to 8 years of education; 61 by participants with 9 to 14 years of education; and 74 by participants with 15 years or more of education.

Besides excluding descriptions, minor modifications in the texts used in this study were made: commas were included in lists of topics, and full-stops before capitalization or at the end of descriptions. These modifications were carried out so that AIC could perform better and calculate features correctly, functioning according to the human analysis in Toledo.^18^ Table 1 shows two examples of descriptions (an example for each picture described) before and after the addition of punctuation. All misspelled words in Brazilian Portuguese are underlined in the examples. The number of misspellings and lack of punctuation in descriptions by individuals with 3 to 8 years of education was significant.

**Table 1:** Examples of original and edited descriptions

	Original examples	Edited examples
30-60 (3-4 years of education) Simple Picture	Eles estão vendo televisão/They are watching television	Eles estão vendo televisão./They are watching television.
	Ele esta lendo jornal/He is reading a newspaper	Ele esta lendo jornal./He is reading a newspaper.
	Eu veno um cachoro/I see a dog	Eu veno um cachorro./I see a dog.
	Eu esto vendo uma casa/I am seeing a house	Eu esto vendo uma casa./I am seeing a house.
30-60 (5-8 years of education) Complex Picture	Eu estou vendo vários carros com motorista pessoas nas janelas	Eu estou vendo vários carros com motorista, pessoas nas janelas.
	Moco taocado pneu mulher andado/I am seeing several cars with drivers people in the windows	Moco taocado pneu, mulher andado./ I am seeing several cars with drivers, people in the windows.
	Young man changing tire woman walking	Young man changing tire, woman walking.

Table 2 shows some statistics concerning the corpus of descriptions used in the experiments, stratified by classes of years of education.

**Table 2:** Statistics from the corpus of descriptions.

Education	# Words Mean values (SD)	# Sentences Mean values (SD)	Clauses/Sentences Mean values (SD)	Writing time Mean values (SD)	# Descriptions
3-4 years	16.5 (8.32)	1.91 (1.25)	2.01 (1.63)	6.42 (4.51)	43
5-8 years	28.4 (17.8)	2.14 (1.36)	3.00 (2.33)	5.08 (2.46)	64
9-15 years	26.1 (12.4)	2.08 (3.04)	3.08 (1.95)	4.27 (2.17)	61
15+ years	48.1 (29.4)	3.58 (2.66)	2.86 (2.52)	3.64 (2.16)	74

SD: standard deviation.

**Features description.** Our set of features (Table 3) is composed of three groups. The first has 46 cognitively motivated features (features 1-46), which are derived from Coh-Metrix-Port.

**Table 3:** Set of features for all experiments.

1. number of words (LE)	39. incidence of adverb ambiguity (SE)
2. number of sentences (LE)	40. argument overlap in adjacent sentences (DI)
3. words per sentence (LE)	41. argument overlap in previous sentences of the text (DI)
4. syllables per word (LE)	42. word stem overlap in adjacent sentences (DI)
5. verb incidence (MO)	43. word stem overlap in previous sentences of the text (DI)
6. noun incidence (MO)	44. content word overlap in adjacent sentences (DI)
7. incidence of adjectives (MO)	45. anaphor reference in adjacent sentences (DI)
8. incidence of adverbs (MO)	46. anaphor reference in previous sentences of the text (DI)
9. incidence of pronouns (MO)	47. ratio of number of simple words to number of words (LE)
10. incidence of content words (verbs, nouns, adjectives, and adverbs) (MO)	48. ratio of number of sentences in passive voice to number of sentences (SI)
11. rlesch Index for Portuguese (LE)	49. ratio of number of sentences initiated by subordinate conjunctions to number of sentences (SI)
12. syllables per word (LE)	50. ratio of number of sentences initiated by coordinate conjunctions to number of sentences (SI)
13. occurrence of noun phrases (SI)	51. mode of the number of clauses (SI)
14. occurrence of modifiers per noun phrase (SI)	52. ratio of average number of clauses to number of sentences in the text (SI)
15. occurrence of words before main verbs (MO)	53. ratio of number of coordinate conjunctions to number of words (SI)
16. frequency for content words (MO)	54. ratio of number of subordinate conjunctions to number of words (SI)
17. minimum frequency for content words (MO)	55. ratio of number of gerunds to number of verbs (SI)
18. incidence of all connectives (LE)	56. ratio of number of participles to number of verbs (SI)
19. incidence of positive additive connectives (LE)	57. ratio of infinitives to number of verbs (SI)
20. incidence of negative additive connectives (LE)	58. ratio of total of gerunds, participles and infinitives to number of words (SI)
21. incidence of positive temporal connectives (LE)	59. ratio of average number of preposition phrases to number of sentences in the text (SI)
22. incidence of negative temporal connectives (LE)	60. ratio of average number of preposition phrases to number of clauses in the text (SI)
23. incidence of positive causal connectives (LE)	61. ratio of number of relative clauses to number of verbs (SI)
24. incidence of negative causal connectives (LE)	62. ratio of number of restrictive appositives to number of sentences (SI)
25. incidence of positive logical connectives (LE)	63. ratio of number adverbial adjuncts to number of sentences (SI)
26. incidence of negative logical connectives (LE)	64. ratio of number of personal pronouns to number of words (LE)
27. incidence of logical operators (LE)	65. ratio of number of possessive pronouns to number of words (LE)
28. incidence of number of *"e" *("and") (LE)	66. ratio of total number of markers to number of words (LE)
29. incidence of number of *"ou" *("or") (LE)	67. ratio of total number of ambiguous markers to number of markers (LE)
30. incidence of number of *"se" *("if") (LE)	68. description time (TA)
31. incidence of number of negations (LE)	69. simple or complex description (TA)
32. incidence of personal pronouns (LE)	70. amount of information (TA)
33. incidence of pronouns per noun phrase (SI)	71. understood the main information (yes/no) (TA)
34. incidence of type/token ratio (LE)	72. age (TA)
35. incidence of verb hypernym (SE)	73. picture presentation order (TA)
36. incidence of verb ambiguity (SE)	74. percentage of misspellings (LE)
37. incidence of noun ambiguity (SE)	75. percentage of positive words from the LIWC dictionary (SE)
38. incidence of adjective ambiguity (SE)	76. percentage of negative words from the LIWC dictionary (SE)

LE: use of lexicons or sentence segmentation tools; MO: use of morphosyntactic taggers; SI: use of full or shallow parsers; SE: use of semantic dictionaries, thesauri, WordNets; DI: use of tools for discourse evaluation; TA: use of features dedicated to the task, whose processing was not manually calculated. Incidence corresponds to the number of units classified for a given measure divided by the number of total words in the text by 1,000 words.

The second group has 21 features: one feature (47) uses Biderman's^28^ dictionary of child and youth words to calculate the percentage of more frequent/common (and consequently more simple) words from the descriptions; 16 features are derived from the parser Palavras^29^ (features 48-63), which help to retrieve the "syntactic skill" feature used by Toledo;^18^ and four lexical features about the use of pronouns and connectives (features 64-67).

The last group contains six features derived from Toledo,^18^ which were not extracted automatically (features 68-73), for they are related to the picture themes. There are three features (features 74-76) especially developed for the description task. One of them uses the Unitex-PB dictionary^30^ to calculate the percentage of misspellings. The last two features use the LIWC dictionary (http://www.liwc.net/), developed for analyzing feelings and opinions. The LIWC dictionary has been translated into BP (*cf*. details in Balage et al.).^31^

The features shown in Table 3 were also classified according to the NLP tools or resources required for their extraction.

**Machine learning and feature selection methods.** The Weka package was used in all experiments to train classifiers and select features.^32^ Six methods that represent different ML approaches were included based on statistics, trees, neural networks, maximum entropy, and rules. The methods used were: Support Vector Machine (SVM) with a radial basis function (RBF) kernel; Naïve Bayes; J48 (implementation of the C4.5 decision-tree algorithm); Multilayer Perceptron (MLP); Logistic Regression (maximum entropy algorithm, called SimpleLogistic in Weka); and JRip (implementation of Repeated Incremental Pruning to Produce Error Reduction – RIPPER - in Weka).

When training ML classifiers on a high-dimensional training set (one that has a relatively large number of attributes, which is the case here), it is important to use *feature selection*. Such methods try to pick from the many features available, those that better separate the classes, and therefore have the most impact on the classification task. The following feature selection algorithms were used:

(1) ranking-based selection;
(2) correlation-based feature selection (CFS); and
(3) manual selection.

The ranking-based method used is an intersection of the results from two other ranking methods: one is based on *information gain* (InfoGainAttributeEval in Weka) and the other on SVMs (SVMAttributeEval). The first method lists attributes based on their information gain, while the second trains an SVM classifier using each attribute and lists them based on the classification performance achieved.

The second method used (CFS) evaluates the quality of a sub-set of attributes based on the correlation between each attribute and the class, and on the correlation of the attributes among themselves.^33^

In the third method (manual selection) we attempted to select the attributes that best represented the impact variables adopted by Toledo.^18^ This manual selection was intended to be compared with automatic methods, and resulted in 21 chosen attributes.

In all of the three sets of experiments carried out, 10-fold cross-validation was performed; the performance measure used was the F-measure. The majority classes in each experiment of the three sets were used as baselines. For experiments 4, 5, and 6, reported in Table 4, a statistical significance test was conducted – a paired two-tailed t test, with a confidence of 0.05 – on F-measure, using the leave-one-out approach to evaluate which classifiers were statistically better.

**Table 4:** Performance of classification methods to evaluate classification difficulty involving the intermediate classes 5-8 and 9-15.

Algorithm	Exp. 1	Exp. 2	Exp. 3	Exp. 4	Exp. 5	Exp. 6
NaïveBayes	70.9	74.2	75.7	81.3	74.2	80.4
SVM	43.9	68.0	**97.7**	**97.7**	**97.7**	**97.7**
MLP	69.0	81.7	93.5	93.2	91.6	93.5
SimpleLogistic	73.4	86.7	86.3	85.2	81.8	84.8
JRip	**73.6**	**87.9**	88.6	86.3	87.8	88.3
J48	66.2	79.3	93.2	91.2	90.5	92.8
Baseline	**59.1**	**75.8**	**51.1**	**51.1**	**51.1**	**51.1**

## RESULTS AND DISCUSSION

Initially, we attempted to train a classifier using the four classes of Toledo^18^ (3 to 4, 5 to 8, 9 to 15, and 15+ years of education), but this did not produce good results. The best classifier was the MLP, with an F-measure of 42.3%. Thus, three sets of experiments were designed with the edited texts, all with binary classifiers, so that the classes have a larger number of examples. This also allows for future refining with a top-down hierarchical approach such as that carried out by Maziero and Pardo^33^ to distinguish the union of classes containing two neighboring groups of years of education from Toledo.^18^

The first set uses two classes (3-8 *versus* 9+ years of education) to evaluate whether the completion of mandatory education represents a significant boundary in the written expression of individuals. Four experiments were conducted using the six ML methods. In Experiment 1, which uses all 76 attributes, the method with the best F-measure was SimpleLogistic (69.8%). Experiment 2 showed that NaïveBayes had a better performance (71.8%); our ranking method was applied to select 23 features, 8 appearing in the 21 attributes of the manual method. The 21 features of the manual method resulted in SimpleLogistic as the best performing classifier in Experiment 3 (71.3%) and in Experiment 4 (71.2%), where CFS was used to select 7 features. The results of these experiments prove our hypothesis that it is possible to use automatic feature selection methods to generate classifiers that perform similarly to those that use manual feature selection. However, the results from the first set of experiments did not exceed an F-measure of 72%. Therefore, the expected boundary at nine years of education to divide classes did not correspond to a boundary for better performance.

The second set of experiments uses the division of extreme classes (3-4 *versus* 15+) in Exp. 1 and intermediate classes (5-8 *versus* 9-15) in Exp. 2 to Exp. 5. In Exp. 1 and Exp. 2, all features were used; in Exp. 3 to Exp. 5, the attribute selection methods were used.

Exp. 1 has SimpleLogistic (84.6%) as the best performing classifier. Exp. 2 also used all features to separate classes 5-8 from 9-15, without success (59%). In Exp. 3, the same 21 manually-selected features as the first set were employed, with SimpleLogistic emerging as the best classifier (67.2%). Exp. 4 and Exp. 5 represent an attempt to improve the performance of manual selection. In Exp. 4, our ranking-based feature selection was applied, and the best result was achieved by JRip (63.2%).

Exp. 5 was interesting in that it resulted in a set chosen by CFS with only one feature (frequency of content words). This feature is not listed in the manual selection of automatic attributes, although it has been selected by Weka's ranking methods. This indicates it may be an option to include this feature in patients' clinical evaluations, since it requires only a morphosyntactic tagger to count grammatical classes. Exp. 5 resulted in the best performance for separating the 5-8 from the 9-15 class; JRip (71.2%).

The third and last set of experiments evaluates which class (or both) from the original experiments in Toledo^18^ may be causing the poor performance of classifiers with 5-8 and 9-15 classes. Table 4 shows the last three experiments for intermediate classes. In these experiments, it was necessary to balance datasets to obtain a higher F-measure.

Exp. 1 excludes the 9-15 class. JRip has the best performance (73.6%). Exp. 2 also has JRip as the best classifier in the division between the 3-4 and 9+ classes (87.9%). Exp. 3, in which instances were duplicated to create a more balanced dataset, had the best classifier among the testing sets and suggests that the 5-8 class may be gathering description specimens that are characteristic of both the 3-4 class and the 9-15 class. Therefore, we suggest that a way to carry out future experiments with descriptions by healthy individuals as controls would be to reclassify the 5-8 class descriptions into the 3-4 and 9-15 classes, using feature selection classifiers for the balanced 3-4 and 9+ classes.

In Exp. 4, SVM showed the best performance when our ranking-based selection method, which selected 26 features, was used. Seven features selected in this experiment (number of words, Flesch Index, writing time, amount of information, coordinate conjunctions, misspellings, words per sentence) also appear in the 21 attributes of the manual selection. These 21 features resulted in the SVM as the best performing classifier in Exp. 5. Exp. 6 used CFS, which selected 21 features, eight of which belong to the set of 21 manually-selected features. The best classifier is SVM, which performed similarly in experiments 3 to 6, with an F-measure of 97.7%.

To select the best classifier in this two-class scenario, a paired two-tailed t-test was conducted. The methods used in Exp. 3, 4, and 5 of Table 4 were compared with SVM. In Exp. 3, 4, and 5 (Table 4), there is no difference between SVM, MLP, JRip, and J48. Based on the hit rate, and not the F-measure, SVM was the best of all, with 100% in all three experiments. Therefore, the SVM with an RBF kernel is the most recommended approach for the binary classification of our data, classifying three of the four initial classes and answering the research question (i).

With an equal or better performance, when compared with other selection methods, it can be concluded that CFS is a strong candidate to replace manual feature selection, answering the research question (ii).

Our investigation reported in Cunha et al.^26^ is pioneering in applying an automated method to BP with clinical purposes. It highlights levels of education, an important variable in the Brazilian scenario, and adapts constructs to study healthy Brazilians who serve as a reference for the study of brain-injured subjects, without missing the opportunity to interact with researchers from other languages and cultures.

The analysis was only possible because it was based on sections of discourse, which represent the whole of subjects' responses. Had isolated sentences or words been analyzed, the same results would not have been achieved. We observed that the analyses performed by Coh-Metrix-Port and AIC acted on texts, thereby calculating the features of a given discourse, albeit produced now by healthy individuals with different years of education or in the future by brain-injured patients, whose transcripts will be compared with those of healthy controls in the Coh-Metrix-Dementia environment.

We succeeded, in this first study using BP, in identifying the classifier with the best F-measure, separating the 3-4 class from the 9-15 class. With regard to the set of features, the three groups of experiments showed that part of the manually selected features is retrieved by automatic selection methods in their search for the features that present higher discriminative power. Therefore, manual selection can be replaced in the future when building classifiers for evaluating brain-injured patients.

We have not yet achieved good results for the four classes of years of education from Toledo,^18^ but our results corroborate the data from the 2012 INAF report. The data emphasize that 59% of those who complete at least one grade of the second cycle of elementary education reach the basic level of literacy, making it difficult to define a cohesive class for individuals with 5-9 years of education.

Our results may have been influenced by the discourse type. To this end, Armstrong^34^ suggests text length and combinations of discourse genres according to the objective to be achieved. Although we have two tools that bring together more than 70 features, there are several studies in the literature that use Idea Density, a complex measure to evaluate transcribed speech. Chand et al.^35,36^ have designed a manual and a rubric to operate this measure, allowing comparison with the features extracted by the tools used in the present study.

## CONCLUSIONS AND FUTURE WORK

We conclude that an SVM with an RBF kernel is the most recommended approach for the binary classification of our data and that CFS is a strong candidate to replace manual feature selection methods, allowing for clinical studies that will be faster, richer in features, and more diverse. The first recommendation for future studies is redimensioning the evaluation, adding other discourse types, since the description task was difficult to separate into classes of years of education, given the need to remove a class from Toledo^18^ to obtain a high-performance classifier. When Coh-Metrix-Dementia is ready, speech transcripts of populations diagnosed with linguistic-cognitive disorders and dementia can be evaluated.
